# Performance of National Measles Case-Based Surveillance Systems in The WHO African Region. 2012 – 2016

**Published:** 2018-08-02

**Authors:** Balcha Masresha, Reggis Katsande, Richard Luce, Amadou Fall, Messeret Shibeshi, Goitom Weldegebriel, Richard Mihigo

**Affiliations:** 1 WHO Regional Office for Africa, Brazzaville, Congo; 2 WHO Inter-country Support Team for Central Africa, Libreville, Gabon; 3 WHO Inter-country Support Team for Western Africa, Ouagadougou, Burkina Faso; 4 WHO Inter-country Support Team for East and Southern Africa, Harare, Zimbabwe

**Keywords:** Case Based Surveillance, Acute Flaccid Paralysis, WHO African Region, Measles, National Systems

## Abstract

Case based surveillance for measles is implemented in the African Region integrated with Acute Flaccid Paralysis (AFP) surveillance. In 2011, the Region adopted a measles elimination goal to be achieved by 2020, which included coverage, incidence and surveillance performance targets. We reviewed measles case-based surveillance data and surveillance performance from countries in the African Region for the years 2012 - 2016. During this period, a total of 359,019 cases of suspected measles were reported from the 44 of 47 (94%) countries using the case based surveillance system. Of these, 202,126 (56%) had specimens collected for laboratory testing. A total of 39,806 measles cases and 25,679 rubella cases were confirmed by IgM serology. Twelve countries met the two principal surveillance performance indicators for each year during the period and four countries met neither indicator over the period. At the Regional level, both surveillance targets were met in 3 of the 5 years in the period of study; however performance varies widely by country. Surveillance performance did not improve across the Region during the 5 years period. High quality surveillance performance is critical to support the achievement of the regional measles elimination goal. Better integrating implementation with AFP surveillance, securing predictable long-term funding sources, and conducting detailed evaluations at country level to identify and address the root cause of performance gaps is recommended.

## Introduction

The 47 Member States of the African Region of the World Health Organization established a goal in 2011 to achieve measles elimination by 2020 using the following strategies: attaining high routine immunization coverage; conducting measles supplemental immunization activities (SIAs); conducting case based surveillance with laboratory confirmation of suspected cases and improving management of measles cases.

The targets for measles elimination^a^ are 1) ≥95% coverage with the first dose of measles-containing vaccine (MCV1) at national level and in each district 2) ≥95% supplemental immunization activity (SIA) coverage in every district, 3) confirmed measles incidence of <1 per million population in all countries, and 4) attaining the targets for the two principal surveillance performance monitoring indicators which are: ≥80% of districts with ≥1 suspected measles case with blood specimen reported per year and a non-measles febrile rash illness rate of ≥2 per 100,000 population^1^.

Countries in the African Region started implementing measles case-based surveillance just before or immediately after their initial wide age range measles supplemental immunisation activities (reaching children between 9 months and 14 years of age), when the burden of measles cases sharply declined. The case based surveillance system was established using the infrastructure available for polio surveillance and according to the Regional guidelines for integrated disease surveillance and response (IDSR)^2^. In case-based surveillance system, each suspected measles case is investigated including laboratory testing. The case definition of a suspected measles is generalized maculopapular rash and fever plus one of the following: cough, coryza (runny nose), or conjunctivitis. For each suspected measles case, an individual case investigation form is completed and a blood specimen collected and sent to the national laboratory for testing for measles-specific immunoglobulin M (IgM) antibody. If the laboratory result is negative for measles IgM testing, then it is tested for rubella IgM^3^.

A confirmed case of measles is one that has had positive serological confirmation of measles-specific IgM antibody in a person who had not received measles vaccination within 30 days before the specimen collection, or one with epidemiological linkage to a lab confirmed case of measles during a confirmed outbreak period, or a case of suspected measles that is clinically compatible but was not investigated with a lab specimen or linked to an outbreak^3^.

A case of measles confirmed by epidemiological linkage is a suspected measles case that has not had a specimen taken for serologic confirmation and is linked (in place, person and time) to lab confirmed cases; i.e., living in the same or in an adjacent district with a lab confirmed case where there is a likelihood of transmission; onset of rash of the two cases being within 30 days of each other.

At the Regional level, the performance of the case based surveillance system is regularly monitored using standard performance indicators which assess the sensitivity of the case detection and investigation system, but also the geographic spread of case notification and investigation. National surveillance programmes are encouraged to regularly monitor the performance of their surveillance system to ensure high quality performance and surveillance sensitivity.

Forty four of the 47 countries in the Region have case based surveillance systems, with Seychelles, Mauritius and Sao Tome being the exceptions. Nigeria and Ethiopia have 4 and 3 measles serology laboratories respectively, while each of the remaining countries in the surveillance network are supported by one national measles serology laboratory that conduct IgM testing of blood specimens using a standard test protocol and tools. All of these national laboratories undergo external quality assurance and accreditation exercises, coordinated by the Regional office of the WHO^4^.

In the case of large laboratory-confirmed outbreaks, countries use line lists that include fewer epidemiological variables and the information is sometimes sent through a parallel reporting system, and the data are captured using a system of aggregate reporting, which lacks details to allow accurate final case classification, or the measurement of surveillance performance Countries report the number of confirmed measles and rubella cases to the WHO and UNICEF officially at the end of the year, using the Joint reporting form (JRF)^5^. This report should ideally match the data in the case based surveillance databases.

## Methods

Case-based surveillance data are compiled at the national level into a computerized database that is shared with WHO on a weekly basis. We analysed the case-based surveillance data for the 5 years period from 2012 to 2016, to determine the quality of surveillance performance.

Surveillance performance is measured using standard indicators, of which two are considered principal monitoring indicators^b^. The non-measles febrile rash illness rate measures the level of case finding and investigation taking place in countries. The proportion of districts investigating suspected measles cases with blood specimen attempts to measure the representativeness of all subnational administrative units in the case investigation efforts. Other indicators of the quality of field surveillance include the following; the proportion of suspected measles cases investigated; the interval between notification and investigation of suspected cases; the interval between the shipment of measles specimens from the field to the lab and the time of receipt at the lab; the proportion of measles outbreaks investigated with blood specimens from the first five cases; the proportion of measles outbreaks with documentation done on measles viral strains.

This analysis only focused on the case detection and investigation component, and did not include the laboratory performance indicators.

## Results

In the years 2012 and 2013, a total of 43 countries were part of the surveillance network. Since 2014, South Sudan joined the African regional surveillance network as the 44^th^ country in the Region.

In the 5 years between 2012 and 2016, a total of 359,019 cases of suspected measles were reported from the 44 countries in the network, out of which 202,126 (56%) specimens were collected for processing at the national laboratories. The average number of specimens collected from suspected measles cases across the Region was 40,425 per year. The largest number of suspected and confirmed cases of measles was reported in 2013. The laboratory testing confirmed 39,806 measles cases and 25,679 rubella cases by IgM serology. Additional measles case confirmation was done by epidemiological linkage and clinical compatibility for a total of 200,027 confirmed cases in the five years covered in the analysis. (Table 1) The annual incidence of confirmed measles across the Region ranged between 29.1 and 76.9 cases per million population per year. (Table 2)

The annual rate of non-measles febrile rash illness cases ranged between 2.5 and 3.4 per 100,000 population across the years. In addition, 77% - 84% of the districts in these target countries reported investigating suspected measles cases with a blood specimen per year. The non-measles febrile rash illness rate has been above the minimum target of 2 per 100,000 population at the Regional level throughout the 5 years, while the proportion of districts reporting at least one suspected measles case with a blood specimen fell below the target of 80% in 2013 and 2014. (Table 1)

Over the 5 years period, 27 countries had an average of 2 or more non-measles febrile rash illness rates. In 2016, a total of 25 countries reported a non-measles febrile rash illness rate of 2 or more per 100,000 population. (Table 3) There was no significant change in the proportion of districts reporting across the years, with an average of 16 countries not achieving the 80% target in each of the five years.

Twelve countries met both surveillance performance targets in each of the five years. These are Cameroon, Congo, Ghana, Lesotho, Mozambique, Rwanda, Senegal, South Africa, Swaziland, Togo, Uganda, Zimbabwe. On the other hand Algeria, Burundi, Cape Verde, Chad, Comoros, Guinea Bissau, Liberia, Malawi, and Tanzania had non-measles febrile rash illness rate of less than 2 per 100,000 population in all five years under review. The target of 80% districts reporting was missed in all five years in Algeria, Angola, Burundi, Cape Verde, Eritrea, Guinea Bissau, Mauritania and Zambia.

The timeliness of arrival of samples from the field to the laboratory has shown a decline from 72% in 2012 to 67% in 2016 (Table 1).

## Discussion

Measles surveillance in the African Region is modelled after Acute Flaccid Paralysis (AFP) surveillance, and is implemented using the resources and infrastructure that was established for AFP surveillance^6^. The current measles case based surveillance system and the case classification scheme was developed in 2002 in order to support countries to document the disease burden and the change in epidemiological patterns soon after the wide age range measles SIAs. Since then, many countries have improved their routine immunisation coverage, and implemented numerous follow up SIAs at intervals of 2 – 4 years, depending on the routine immunisation coverage, and the country-specific epidemiological context^7^, ^8^.

The number of suspected measles cases reported annually from 2012 – 2016 have increased significantly as compared to the reports in 2009^9^. This is due to an increase in the number of reporting countries from 40 in 2009 to 44 in 2016. In addition, large measles were outbreaks were reported from Nigeria in 2013, with a total of 52,900 cases.

This analysis reveals that, since the adoption of the measles elimination goal in 2011, the quality of measles surveillance has not shown significant improvement in the Region. The performance at Regional level shows that the targets for the two principal monitoring indicators were met in 2012, 2015 and 2016. In addition, there are major gaps in individual country performance, with less than two thirds of the countries meeting the targets for both principal monitoring indicators.

AFP surveillance activities in the Region are well funded and supported by a network of dedicated staff. The latest published report on AFP surveillance performance for the African Region (data up to 8 November 2016) shows that only 7 out of the 47 countries failed to reach the target for the annualised non-polio AFP detection rate, while only 4 countries had less than 80% adequate specimens^10^. Hence, countries can do more in terms of ensuring better measles case detection and reporting by reinforcing the integration into AFP surveillance activities. However, with the winding down of the global polio eradication program, and the transition of polio assets, there is a serious risk that measles surveillance will remain under resourced.

The decline in the number of countries that achieved 80% availability of timely laboratory results was partly due to the stock-out of serological test kits experienced by the network laboratories in 2015 and 2016.

The number of measles cases officially reported by countries to the WHO and UNICEF indicate discrepancies with the number of cases confirmed through the case based surveillance system in the Democratic Republic of Congo^5^. This is due to the heavy reliance on aggregate reporting of measles in the country instead of using the case-based surveillance system, and so leads to an under-representation of the true magnitude of measles in the case based surveillance database.

As the Region makes progress towards the measles elimination targets, a more rigorous implementation of disease surveillance will be needed to identify each and every suspected case and cluster of cases^8^. Investigation of measles outbreaks requires that nasopharyngeal swabs specimens be collected from confirmed cases for genotyping of circulating viral strains. As countries make progress towards elimination, genotyping analysis will be more important in determining the origin and spread of measles viruses, and to document the interruption of chains of transmission^11^.

While the Region has made significant progress in reporting rubella cases and documenting its burden using the existing surveillance system, there is a need to broaden the scope of surveillance to “fever and rash surveillance” in order to be able to capture all suspected measles as well as suspected rubella cases. The WHO Regional Office has developed guidelines to support the implementation of a more sensitive and rigorous fever and rash surveillance system, that responds to the demands of the elimination program. However, implementing this elimination-standard surveillance requires additional resources at all levels^4^.

Disease surveillance provides the critical information needed to quickly detect and stop transmission of measles, to identify immunity gaps and fix areas of programme weakness, and to drive immunisation programme activities. In addition, surveillance plays a critical role in documenting the progress towards elimination and in the eventual verification of elimination at country and Regional level. When determining whether a country has achieved elimination, the verification exercise considers 5 lines of evidence – disease epidemiology, population immunity, quality of surveillance, sustainability of the programme, and genotyping evidence – to allow for a comprehensive evidence-based assessment of past programme performance and future capacity to sustain elimination^12^.

In conclusion, measles surveillance performance in the WHO African Region has not shown significant improvement between 2012 and 2016. The root causes of this performance gap need to be explored in the priority countries and addressed on a county by country basis. Exploring opportunities for strengthening the integration of measles surveillance with AFP surveillance activities could help to optimise the utilization of resources and improve sensitivity. Countries and partners should provide the necessary programmatic attention and long-term investments to strengthen measles surveillance and laboratory activities, in light of the elimination targets, and the expected decline in polio resources in the coming years.

This analysis looked only at the performance of the measles case-based surveillance system, and did not include epidemiological analysis of reported cases. We did not include detailed review of data from aggregate reporting systems. Moreover, our analysis only focused on the case detection and investigation component, and did not include laboratory performance or the quality of outbreak investigation.

## Figures and Tables

**Table 1:** Measles surveillance performance. African region. 2012 - 2016.

Parameter	2012*	2013*	2014	2015	2016
Total suspected measles cases reported	55717	101196	71566	68769	61771
Number of cases with specimens collected	39693	41920	42690	38038	39785
% suspected measles cases investigated	91%	78%	85%	82%	86%
% lab results available	96%	88%	88%	83%	83%
Measles cases confirmed by lab	8920	9831	8329	6717	6009
Measles cases confirmed by lab, epidemiological linkage and clinically	25609	69910	37847	37838	28823
Rubella cases confirmed by lab	6659	3918	6106	5546	3450
Non-measles febrile rash illness rate (NMFRI) per 100,000 population#	3.4	2.9	3.0	2.5	2.5
Number of countries meeting the 2 per 100,000 target for NMFRI rate	30/43	25/43	26/44	27/44	25/44
Proportion of districts reporting at least 1 suspected case with blood specimen#	84%	78%	77%	82%	82%
Number of countries meeting the 80% target for proportion districts reporting	26/43	27/43	28/44	24/44	28/44
Number of countries with at least 80% lab results available	40/ 43	35/43	34/44	37/44	34/44
% specimens received at the lab within 3 days of collection	49%	47%	51%	48%	36%
% specimens received at the lab within 7 days of collection	72%	69%	71%	68%	67%

# Principal surveillance performance indicators

* South Sudan joined the African Region of the WHO in late 2013, and so the denominator for 2012 and 2013 is 43 countries, while for more recent years, the denominator is 44 countries.

**Table 2:** Incidence of confirmed measles in AFR. 2012 - 2016

	2012	2013	2014	2015	2016
Incidence of confirmed measles per million population	30.2	76.9	39.9	39.4	29.1
Number of countries with incidence levels of < 5 per million	18/43	22/43	21/44	25/44	22/44
Number of countries with incidence levels of < 1 per million	12/43	15/43	12/44	14/44	11/44

**Table 3:** Number of countries according to their surveillance performance. 2012 - 2016

NMFRI rate per 100,000 population	2012	2013	2014	2015	2016
< 0.5	5	7	7	4	3
0.5 - 0.9	2	6	3	3	4
1.0 - 1.9	6	5	8	10	12
2.0 and above	30	25	26	27	25
	**43**	**43**	**44**	**44**	**44**
Proportion of districts reporting at least one suspected case with a blood specimen					
<60%	9	13	13	8	8
60- 69%	3	2	1	6	3
70 - 79%	5	1	2	6	5
80% and above	26	27	28	24	28
	**43**	**43**	**44**	**44**	**44**

## Footnotes

a Measles elimination is defined as the absence of endemic measles transmission in a defined geographical area (e.g. region or country) for ≥12 months, in the presence of a well-performing surveillance system

b The non-measles febrile rash illness rate, and the proportion of districts investigating suspected measles cases with blood specimen.
